# Utilization of Jumbo-Sized Cups in Conjunction With Dual-Mobility Constructs Does Not Increase Risk of Re-Revision in Revision Hip Arthroplasty

**DOI:** 10.1016/j.artd.2025.101879

**Published:** 2025-10-23

**Authors:** Ramesh B. Ghanta, Jeffrey Barry, Jeffrey Kwong, Hunter Warwick, Erik Hansen, Claudio Diaz-Ledezma

**Affiliations:** Department of Orthopaedic Surgery, University of California San Francisco, San Francisco, CA, USA

**Keywords:** Revision hip arthroplasty, Dual-mobility, Jumbo cups, Total hip arthroplasty, Instability

## Abstract

**Background:**

Dual-mobility (DM) articulations are increasingly used in revision total hip arthroplasty (THA) to reduce instability, but their effectiveness in conjunction with jumbo cups is unclear. This study evaluated the risk of all-cause and instability-related re-revision when DM articulations were used with jumbo vs standard cups in revision THA.

**Methods:**

A retrospective review included 199 revision THA patients with DM articulations: 156 with regular cups and 43 with jumbo cups (≥62 mm for females, ≥66 mm for males). Exclusion criteria were cup-cage constructs, cemented DM liners in pre-existing cups, and tumor cases. The primary outcome was re-revision, with focus on instability. Student's *t*-test compared revision rates, and multivariable logistic regression with backward selection was used to assess the relationship between cup size and re-revision risk.

**Results:**

At a mean 4.6-year follow-up, re-revision rates were similar between groups (10.3% regular vs 11.6% jumbo, *P* = .79). Instability-related re-revisions were also comparable (5.1% regular vs 7% jumbo; *P* = .64). Logistic regression demonstrated that jumbo cup utilization was not associated with risk of all-cause revision (*P* = .99) or instability-related re-revision (*P* = .77). However, the number of prior surgeries increased risk for both all-cause (OR: 1.32 [1.07, 1.63], *P* = .009) and instability-related (OR: 1.46 [1.13, 1.87], *P* = .003) re-revisions.

**Conclusions:**

Our results demonstrate satisfactory midterm outcomes in both jumbo and regular cup patients implanted with DM systems. These findings demonstrate that the use of DM liners in jumbo cups does not portend increased risk of re-revision compared to use of DM in regular sized cups.

## Introduction

Instability represents a common cause of failure after revision total hip arthroplasty (THA) [1]. The use of dual-mobility (DM) articulations has become increasingly prevalent to address a primary diagnosis of instability as well as to reduce the risk of dislocation in revisions undertaken for other etiologies [2,3]. However, while some studies have pointed to the utility of DM in reducing the rate of dislocation in revisions [4,5]*,* 2 reports from national registries have challenged these findings [6,7].

In a recent study evaluating dislocations after revision THA, Sonn et al. [8] demonstrated that, regardless of DM use, a reduced “outer femoral head diameter-to-acetabular cup size” (*OH:C*) ratio was predictive of dislocation. Another implant ratio related to acetabular component size is the “head-to-acetabular diameter ratio” (*H:A ratio*), calculated by dividing the inner head diameter by the acetabular component diameter. Prior reports have suggested that dislocations and dissociations occur in patients whose DM implants have an H:A ratio of ≤0.5 [9]. This finding highlights the potential role of large cup size in instability-related revision failures.

A systematic review revealed that instability constitutes the most prevalent etiology for re-revision following the use of large size jumbo cups [10]. These jumbo cups have been used to address large acetabular bone defects with good outcomes and survivorship [[3], [4], [5], [6]]. Initially, jumbo cups were employed with polyethylene liners, but currently, most of the commercially available jumbo cups offer a DM option [11].

While both jumbo cups and DM articulations are frequently employed in revision THA, there is no data regarding their effectiveness when used in conjunction. A previous American Joint Replacement Registry Study evaluating the efficacy of DM constructs in revision THA found higher revision rates compared to ≥36 mm heads. However, the cup size was not included in the analysis, and the authors concluded that their “results may be biased due to unidentified covariates associated with implant selection” [6]. The purpose of this study was to assess the risk of re-revision, with a particular interest in instability, when DM articulations were used in conjunction with large diameter jumbo vs regular cup sizes. We hypothesized that the use of jumbo cups does not increase the rate of re-revisions due to instability compared to regular cup sizes when employing DM.

## Material and methods

Ethical approval was granted by the institutional review board. We performed a retrospective analysis of consecutive revision THA patients at a single tertiary care referral center in whom a DM articulation was implanted between 2012 and 2021. DM constructs were chosen on an individual basis by the surgeon based on both preoperative risk factors (activity level, pathology addressed, etc.) and intraoperative stability testing. Abductor attachment and quality is assessed at the time of surgery by all surgeons, and in cases of severe abductor deficiency, constrained liners are preferentially utilized. Cases involving cup-cage constructs, cemented DM liners in a pre-existing cup, cemented DM liners or cups, and tumor-related cases were excluded. Demographic variables including age, patient sex, body mass index, laterality of procedure, American Society of Anesthesiologists score, Charlson Comorbidity Index, and femoral head size were collected.

Patients were divided into 2 cohorts: regular sized cup and jumbo cup. All cups were uncemented. Jumbo cups were defined as those that had an outer diameter greater than or equal to 62 mm in women and 66 mm in men [[12], [13], [14]]. The primary outcome was the rate of re-revision for any reason. We additionally collected information on the etiology for re-revision, including whether the re-revision was performed for a diagnosis of instability. Multivariable logistic regression was used to assess the relationship between all-cause revision and the use of jumbo cup, accounting for the effects of age, gender, body mass index, Charlson Comorbidity Index, reason for revision, number of prior surgeries, surgical approach, and single vs both component revision. Backward variable selection was applied with a threshold for retention of 0.10; the use of a jumbo cup was specified to be included in every model. Statistical significance was set at *P* = .05. Statistical analyses were performed using R studio (Boston, Massachusetts, USA) and SAS OnDemand for Academics (Cary, North Carolina, USA).

We included 199 patients who underwent revision THA with implantation of a DM construct, with 156 (78.4%) receiving regular cup/DM implantation and 43 (22.6%) receiving jumbo cup/DM implantation. Minimum follow-up for included patients in the study was 2 years. Demographic characteristics are summarized in Table 1.

**Table 1:** Patient demographics for regular cup and jumbo cup cohorts.

Demographic variable	Regular cup (n = 156)	Jumbo cup (n = 43)	*P* value
Age (mean ± SD)	64.9 ± 12	65.1 ± 9.8	.92
No. of female patients (%)	89 (57.1%)	22 (51.2%)	.49
Laterality (left/right)	71/85	19/24	.87
Surgical approach (direct lateral/posterolateral)	32/124	6/37	.33
BMI (mean ± SD)	27.8 ± 6.3	29.1 ± 7	.2
No. of ASA3 patients (%)^a^	68 (43.6%)	17 (39.5%)	.63
No. of prior surgeries (mean ± SD)	1.1 ± 1.7	1.8 ± 1.4	**.01**

BMI, body mass index.

Bold values indicate statistical significance.

a American Society of Anesthesiologists score.

**Figure 1:**
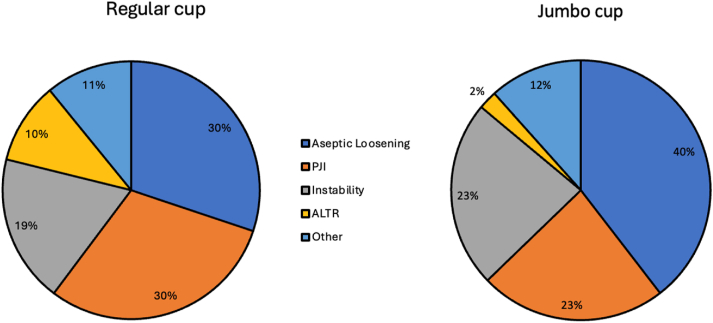
Initial indication for revision hip arthroplasty in regular cup/dual-mobility and jumbo cup/dual-mobility cohorts. PJI, prosthetic joint infection; ALTR, adverse local tissue reaction.

The primary indication for revision THA was identified from the chart review. Aseptic loosening was the most common reason for revision, and instability the third most common reason in both cohorts (Fig. 1). Patients in the jumbo cup/DM cohort had a mean of 1.8 surgeries performed between index arthroplasty and the time of revision with DM implantation; by contrast, the patients who had regular cup/DM implanted had a mean of 1.1 surgeries (*P* = .01). In the jumbo cup/DM cohort, 58.1% of patients underwent revision of the cup and femoral stem. The remainder had revision of the cup alone. In the regular cup/DM cohort, 53.2% of patients underwent revision of the cup and stem. The median outer diameter of the implanted cup was 66 mm (IQR: 62-70 mm, range: 62-80 mm) in the jumbo cup/DM group and 58 mm (IQR: 54-60 mm, range: 48-64 mm) in the regular cup/DM group. The inner femoral head size was 28 mm in all jumbo cup/DM patients. In the regular cup/DM cohort, 28 mm femoral heads were implanted in 88.5% of patients; the remaining patients had 22 mm heads. The median outer diameter of the femoral head was 52 mm (IQR: 48-58 mm) for the jumbo cup/DM cohort and 46 mm (IQR: 42-48 mm) for the regular cup/DM cohort. The *OH:C* and *H:A ratios* were calculated. Their associations with re-revision and failure due to instability were evaluated.

For the jumbo cup/DM cohort, the cup manufacturer was Stryker in 86% of cases, Smith and Nephew in 9.3%, and Depuy in 4.7%. In the regular cup/DM cohort, the cup manufacturer was Stryker in 87.8% of cases, Smith and Nephew in 6.4% of cases, Depuy in 5.1%, and Zimmer Biomet in 0.6%. Augments were used in 7% of the jumbo cup/DM group and in 6.4% of the regular cup/DM cohort.

## Results

At a mean follow-up of 4.6 years (range: 2-9.5 years), jumbo cup/DM patients had a total of 5 (11.6%) re-revisions vs 16 (10.3%) re-revisions in the regular cup/DM cohort (*P* = .79). Re-revision rate for noninfectious etiologies was 11.6% in the jumbo cup/DM group and 7% in the regular cup/DM patients (*P* = .27).

**Figure 2:**
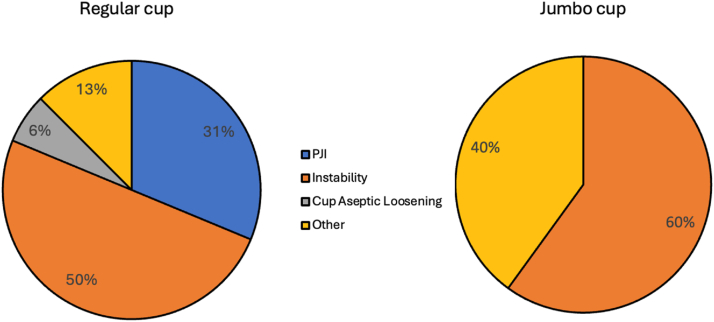
Indication for re-revision in regular cup/dual-mobility and jumbo cup/dual-mobility cohorts. PJI, prosthetic joint infection.

Re-revision rate for instability was 7% (3 cases) in the jumbo cup/DM cohort and 5.1% (8 cases) in the regular cup/DM group (*P* = .64). In both groups, instability was the predominant reason for re-revision. Of note, 1 patient in the regular cup/DM cohort sustained an intraprosthetic dissociation during attempted closed reduction, requiring open reduction. All patients undergoing re-revision for dislocation had 28 mm DM inner heads implanted. Indications for re-revision in both cohorts are depicted in Figure 2.

When stratifying by gender, the female jumbo cup/DM re-revision rate for instability was 9.1% compared to 5.6% in the regular cup group (*P* = .54) while for males, the jumbo cup/DM re-revision rate for instability was 4.8% compared to 3.0% (*P* = .72).

The *OH:C* ratio in the jumbo cup/DM group was 0.77 (IQR: 0.76-0.83) with a significant difference compared to the regular cup/DM group: 0.79 (IQR: 0.77-0.86) (*P* = .04). The OH:C ratio had no significant difference between patients who were re-revised compared to those who were not re-revised (0.79 (IQR: 0.75-0.8) vs 0.79 (IQR: 0.77-0.86), *P* = .09). Also, no difference was found in patients who were re-revised due to instability compared to those who were not re-revised due to instability (0.8 (IQR: 0.76-0.82) vs 0.79 (0.77-0.86, *P* = .8)

The *H:A ratio* in the jumbo cup/DM group was 0.42 (IQR: 0.4-0.45), which was statistically significant compared to the regular cup/DM group, which had a *H:A ratio* of 0.48 (IQR: 0.46-0.5) (*P* < .001). The *H:A ratio* was not different between patients who were re-revised compared to those who were not re-revised (0.45 [IQR: 0.44-0.48] vs 0.47 [IQR: 0.44-0.5], *P* = .1). Also, no difference was found in patients who were revised due to instability vs those who were not re-revised due to instability (0.47 [IQR: 0.44-0.48] vs 0.47 [0.44-0.5], *P* = .3).

Multivariable logistic regression with backward selection was applied to evaluate which variables were associated with the risk for all-cause re-revision. In the final model, the only variable that remained was the number of prior surgeries, in addition to the use of a jumbo cup, which was prespecified to be included in every model. The use of a jumbo cup was not associated with the risk of all-cause re-revision (*P* = .99). However, each prior surgery increased the odds of re-revision (OR: 1.32 [1.07, 1.63], *P* = .009). A similar model was built for instability-related re-revision. Again, the number of prior surgeries (OR: 1.46 [1.13, 1.87], *P* = .003), but not the use of a jumbo cup (*P* = .77), was related to the risk of instability-related re-revision.

We performed additional analyses treating cup size as a continuous variable rather than as a binary categorical variable (regular vs jumbo cup). In these analyses, cup size was similarly not significantly related to either the risk of all-cause re-revision (*P* = .17) or instability-related re-revision (*P* = .35). However, the number of prior surgeries remained significant (*P* = .020 for all-cause revision, *P* = .005 for instability-related re-revision).

## Discussion

The utilization of DM constructs in combination with jumbo cups has emerged as a promising approach to address instability and bone loss in revision THA. However, the efficacy of their combined use remains underinvestigated. This retrospective study encompassed a cohort of patients undergoing revision THA, where DM constructs were employed with either jumbo or standard sized cups. The results of our multivariate analysis revealed comparable revision rates for all causes and specifically for instability between the 2 cohorts, with favorable midterm survivorship. Given the increasing utilization of DM in revision THA, it is crucial to assess any potential impact on instability rates when these bearings are used in conjunction with other implant variables. Cup size is potentially a variable to consider given its indirect association with a higher risk of dislocation after revision THA, as exemplified by Sonn et al.'s description of the *OH:C ratio* [8]. In their experience, a smaller ratio was associated with an increased risk of dislocation in revisions. In our study, we found no differences in re-revision risks in relation to the *OH:C ratio* or the *H:A ratio*.

Two previous national registry studies have demonstrated that the risk of re-revision is not decreased with DM compared to large-diameter femoral heads [6,7]. However, Otero et al [6], did not incorporate cup size into its analysis, acknowledging that their findings may be biased due to undetected covariates associated with implant selection. Additionally, utilizing the Australian Orthopaedic Association National Joint Replacement Registry, Hoskins et al. [7] observed a similar rate of re-revision when comparing DM to large-diameter femoral heads but used 58 mm as a cutoff point to assess the influence of cup size on re-revision rates. Because this categorization does not directly align with the standard definition of jumbo vs regular cup size, it is possible that the relationship between the use of jumbo cups and re-revision may not have been appreciated. Therefore, we attempted to evaluate the impact of a large-size jumbo cup on the performance of DM through a multivariate analysis designed to minimize the effect of potential confounders and found that the use of jumbo vs non-jumbo cups did not influence all-cause rates of re-revision or re-revision for instability rates. It is important to note that these definitions of jumbo and non-jumbo cups are arbitrary values. However, even when performing a supplemental analysis that analyzed cup size as a continuous variable, no difference was identified in revision rates as cup size varied throughout our cohort. The results of this logistic regression analysis further emphasize that cup size does not appear to influence risk of re-revision when using DM in revision THA. Rather, the only identified predictor of re-revision in this cohort was the number of prior surgeries. This finding is likely reflective of the increased complexity of pathology seen in patients with larger cup sizes, who had likely undergone more prior surgeries.

Patients in our jumbo cup/DM cohort had a re-revision rate for instability of 7%, which is similar to rates reported in the literature. In a recent systematic review of jumbo cup outcomes by Wang et al, the authors demonstrated a pooled dislocation/instability revision rate of 5.9% among the 14 included studies [10]. While the majority of the included studies by Wang et al. did not specify whether DM articulations were additionally used, our patient cohort specifically underwent DM implantation, suggesting a higher preoperative risk for instability. Similarly, our cohort of regular cup/DM patients had a re-revision rate for instability of 5.1%, which is also similar to rates reported in literature for patients undergoing DM implantation in the setting of revision THA [15]. We found no difference in re-revision rates between the 2 cohorts despite a higher number of prior surgeries in the jumbo cup cohort, suggesting that DM implantation may improve stability in this subset of revision arthroplasty patients who are at high risk for postoperative complications. Thus, our study appears to demonstrate satisfactory survivorship and optimization of revision THA stability when combining either regular or jumbo cups with DM bearings.

This study has several limitations. First, this study had relatively small sample sizes, owing to the rare nature of concurrent jumbo cup and DM implantation inherent in single-center studies. These small cohort sizes might have rendered the analysis underpowered to detect rates of relatively rare complications, such as revisions. In addition, we did not account for the severity of the pathology that led to the need for revision, which could affect re-revision rates. Differences in implant designs between various companies utilized in each group could also have affected our findings. It is also possible that a mean follow-up of 5 years is insufficient to properly detect the development of recurrent instability, and longer term studies may be needed to demonstrate differences in dislocation rates. Additionally, it is possible that infection can manifest as instability, which alters some of the findings for revision etiologies found in this study. There is also likely a subset of patients who had symptoms of clinical instability but never underwent re-revision and were thus not included in the analysis. Finally, this study describes the experience of our single center, which may restrict generalizability to other similar large academic medical centers.

## Conclusions

We found satisfactory midterm outcomes in both jumbo and regular cup patients implanted with DM systems, with no difference in re-revision rates between the 2 groups. Our results demonstrate that the use of DM liners in jumbo cups does not portend increased risk of re-revision compared to use of DM in regular sized cups.

## Conflicts of interest

J. Barry is a paid consultant for Smith&Nephew, J&J MedTech, Lineage Medical, and Onkos Surgical; and received research support from Smith&Nephew, Omega, and AAHKS. C.D. Ledezma is a member of medical/orthopaedic publications editorial/governing board of *Journal of Arthroplasty*. E. Hansen received royalties from Corin Ltd. and Stryker; and is a paid consultant for Stryker; all other authors declare no potential conflicts of interest.

For full disclosure statements refer to https://doi.org/10.1016/j.artd.2025.101879.

## CRediT authorship contribution statement

**Ramesh B. Ghanta:** Writing – review & editing, Writing – original draft, Investigation, Formal analysis, Data curation, Conceptualization. **Jeffrey Barry:** Writing – review & editing, Writing – original draft, Methodology, Investigation, Formal analysis, Data curation, Conceptualization. **Jeffrey Kwong:** Writing – review & editing, Writing – original draft, Methodology, Investigation, Formal analysis, Data curation, Conceptualization. **Hunter Warwick:** Writing – review & editing, Writing – original draft, Methodology, Investigation, Formal analysis, Data curation, Conceptualization. **Erik Hansen:** Writing – review & editing, Writing – original draft, Methodology, Investigation, Formal analysis, Data curation, Conceptualization. **Claudio Diaz-Ledezma:** Writing – review & editing, Writing – original draft, Methodology, Investigation, Formal analysis, Data curation, Conceptualization.

## Appendix A Supplementary data

Conflict of Interest Statement for Barry

Conflict of Interest Statement for Kwong

Conflict of Interest Statement for Diaz-Ledezma

Conflict of Interest Statement for Ghanta

Conflict of Interest Statement for Hansen

Conflict of Interest Statement for Warwick
