# Supplementary material for: Utilization of Jumbo-Sized Cups in Conjunction With Dual-Mobility Constructs Does Not Increase Risk of Re-Revision in Revision Hip Arthroplasty
Source: Arthroplast Today. 2025 Oct 23;36:101879. doi: 10.1016/j.artd.2025.101879 (PMC12593429; doi:10.1016/j.artd.2025.101879)
Supplement: Conflict of Interest Statement for Hansen [file mmc5.pdf]

Combined CONFLICT OF INTEREST STATEMENT

The Journal of Arthroplasty

(Adopted from the American Academy of Orthopaedic Surgeons disclosure statement)

The following form **must be filled out completely and submitted by each author (example, 6 authors, 6 forms)**. **If no discloser is required, please write/type "none" at the end of each sentence.**

Manuscript Title

Dual Mobility in jumbo cups for revision THA: Winning Combination or a Formula for failure?

1. Royalties from a company or supplier (The following conflicts were disclosed)
   Corin Ltd.; Stryker

2. Speakers bureau/paid presentations for a company or supplier (The following conflicts were disclosed)
   none

3A. Paid employee for a company or supplier (The following conflicts were disclosed)
   none

3B. Paid consultant for a company or supplier (The following conflicts were disclosed) Stryker

3C. Unpaid consultants for a company or supplier (The following conflicts were disclosed)
   none

4. Stock or stock options in a company or supplier (The following conflicts were disclosed)
   none

5. Research support from a company or supplier as a Principal Investigator (The following conflicts were disclosed)
   none

6. Other financial or material support from a company or supplier (The following conflicts were disclosed)
   none

7. Royalties, financial or material support from publishers (The following conflicts were disclosed)
   none

8. Medical/Orthopaedic publications editorial/governing board (The following conflicts were disclosed)
   none

9. Board member/committee appointments for a society (The following conflicts were disclosed)
   none

Each author must sign AND print or type his/her name, date and submit a separate form

In addition, one BLINDED Conflict of Interest form (no author names used) should be submitted per manuscript with all author disclosures.

Erik Hansen, MD	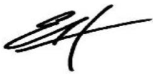	1/13/2025
Author Name (Print or Type)	Author Signature	Date
